# P-1534. The Impact of Broad-spectrum Antimicrobials in Patients with Community-onset Pneumonia at Low Risk of Drug-resistant Pathogens: A Historical Cohort Study

**DOI:** 10.1093/ofid/ofae631.1702

**Published:** 2025-01-29

**Authors:** Takahiro Takazono, Naoki Hosogaya, Yoshiyuki Saito, Masahiko Takemura, Naoki Iwanaga, Noriho Sakamoto, Junichi Hirayama, Rie Ueno, Hiroshi Mukae

**Affiliations:** Nagasaki University Graduate School of Biomedical Sciences, Nagasaki, Nagasaki, Japan; Nagasaki University, Nagasaki, Nagasaki, Japan; Datack, Inc., Tokyo, Tokyo, Japan; Datack, Inc., Tokyo, Tokyo, Japan; Nagasaki University Hospital, Nagasaki, Nagasaki, Japan; Nagasaki University Hospital, Nagasaki, Nagasaki, Japan; BioMérieux Japan Ltd., Tokyo, Tokyo, Japan; BioMérieux Japan Ltd., Tokyo, Tokyo, Japan; Nagasaki University, Nagasaki, Nagasaki, Japan

## Abstract

**Background:**

The administration of broad-spectrum antimicrobials has prevailed in the clinical management of community-onset pneumonia (COP). Growing evidence suggests that the use of unnecessary broad-spectrum antimicrobials for patients with COP leads to adverse events and poor outcomes. This study aims to understand the impact of broad-spectrum antipseudomonal β-lactam use on clinical outcomes and resource utilization in patients hospitalized for COP with a low risk for drug-resistant pathogens (DRPs).

**Methods:**

This historical cohort study analyzed the hospital claims database in Japan from January to December 2018. The study includes patients aged 20 or older hospitalized with COP having intravenous antimicrobial therapy administration and excludes patients with a high risk for DRPs. Patients were selected into a broad-spectrum (antipseudomonal β-lactam therapy) and a narrow-spectrum (non-antipseudomonal β-lactam therapy) group based on the initial antimicrobial regimen. This study evaluates 30-day mortality as a primary outcome by inverse probability of treatment weighting (IPTW)and healthcare resource utilization as one of the secondary outcomes.

**Results:**

A total of 15,617 patients met the inclusion criteria. This target population was divided into two groups: 2,627 patients in the broad-spectrum and 12,990 in the narrow-spectrum group. In the first group, the most commonly used antimicrobials were Piperacillin / Tazobactam (68%), and the 30-day mortality was 10.6 in contrast with 5.3% in the second group. Thus, the broad-spectrum group was associated with an increased 30-day mortality rate in contrast with the narrow-spectrum group after IPTW weighting (adjusted odds ratio 1.77, 95% confidence interval [1.52 to 2.06], < 0.001). This trend was observed regardless of pneumonia severity. The average inpatient cost was $6,139 USD for the broad-spectrum group against $5,184 USD for the narrow-spectrum group, with respective length of stay (LOS) figures of 23.1 and 19.8 days.

**Conclusion:**

This study shows that the initial use of antipseudomonal β-lactams for COP with a low risk for DRPs is associated with higher poor outcomes, including death and higher healthcare resource utilization. Judicious selection of initial antimicrobials is warranted in the management of COP.

**Disclosures:**

**Junichi Hirayama, n/a**, bioMerieux Japan: employee of bioMerieux, salary **Rie Ueno, MD.**, BioMérieux Japan Ltd.: Employee of BioMérieux Japan Ltd. **Hiroshi Mukae, M.D., Ph.D.**, AstraZeneca: Lecture fees|Gilead Sciences: Lecture fees|GSK: Lecture fees|MSD: Advisor/Consultant|MSD: Lecture fees|Pfizer: Lecture fees|Shionogi & Co., Ltd.: Advisor/Consultant|Shionogi & Co., Ltd.: Grant/Research Support|Shionogi & Co., Ltd.: Lecture fees

